# Efficacy of preoperative prophylactic application of betamethasone on postoperative nausea and vomiting in patients undergoing total knee arthroplasty: a prospective randomized controlled trial

**DOI:** 10.3389/fmed.2024.1487818

**Published:** 2024-12-17

**Authors:** Xiaobo Sun, Qunli Dou, Bowei Li, Guoyang Bai, Kai Qin, Jianbing Ma, Fudong Yao, Yuanchi Huang

**Affiliations:** ^1^Department of Knee Joint Surgery, Honghui Hospital, Xi’an Jiaotong University, Xi’an, Shaanxi, China; ^2^The First Clinical Medical College, Shaanxi University of Chinese Medicine, Xianyang, China; ^3^Department of Orthopedics, Baoji Central Hospital, Baoji, Shaanxi, China

**Keywords:** total knee arthroplasty, nausea and vomiting, glucocorticoids, betamethasone, randomized controlled trial

## Abstract

**Background:**

The demand for total knee arthroplasty (TKA) is increasing, yet postoperative nausea and vomiting (PONV) significantly hinder patient recovery. Preoperative prophylactic administration of glucocorticoids can alleviate PONV, with betamethasone showing promising results in breast and cardiac surgeries. However, its efficacy in TKA patients remains unclear. This study evaluates the efficacy and safety of preoperative betamethasone for PONV in TKA patients through a prospective randomized controlled trial (RCT).

**Materials and methods:**

In this trial, 124 patients were randomly assigned to receive either 2 mL of normal saline (control group) or 2 mL of betamethasone sodium phosphate (10.52 mg total dose; experimental group) 10 min before anesthesia induction. Primary outcomes included nausea severity, vomiting frequency, and antiemetic use, while secondary outcomes were pain scores, knee range of motion, blood glucose, IL-6, CRP, ESR, and adverse reactions.

**Results:**

Results showed the experimental group had significantly lower nausea severity at 2, 4, 6, 12, and 24 h post-surgery compared to controls. The average frequency of vomiting in the experimental group (0.060 ± 0.307) was lower than that in the control group (0.390 ± 0.662), with a statistical difference (*P* < 0.001). The postoperative use of metoclopramide in the experimental group (0.480 ± 2.163) was lower than that in the control group (4.520 ± 6.447), and there was a statistical difference between the two groups (*P* < 0.001). CRP in the experimental group on the second day after surgery (45.741 ± 47.044) was lower than that in the control group (65.235 ± 50.970), with a statistical difference (*P* = 0.014). IL-6 in the experimental group was lower on the first (51.853 ± 67.202) and second postoperative days (25.143 ± 31.912) than that in the control group on the first (79.477 ± 97.441) and second postoperative days (38.618 ± 36.282), with statistical differences (*P* = 0.039, *P* = 0.006). There was no significant difference in postoperative knee pain, knee range of motion, blood glucose, ESR, and adverse reactions between the two groups.

**Conclusion:**

Our prospective RCT demonstrates that preoperative betamethasone is effective and safe for reducing PONV in TKA patients, suggesting a new clinical approach for prophylactic treatment of PONV post-TKA.

## 1 Introduction

Knee osteoarthritis (KOA) is a chronic joint disease characterized by articular cartilage degeneration, resulting in knee deformity and pain. Total knee arthroplasty (TKA) is utilized as the last resort in the management of end-stage patients (1). The demand for TKA is expected to increase gradually with the aging population and rising prevalence of obesity (2). TKA can substantially relieve patients’ pain and improve the quality of life, but postoperative nausea and vomiting (PONV) remains a distressing adverse reaction and the second most common complaint following pain. PONV may lead to pulmonary aspiration, hypoxia, electrolyte imbalance, delayed wound healing, etc (3). The occurrence of PONV is related to general anesthesia and intravenous analgesia during surgery (4, 5), as well as inflammatory response (6–9). PONV delays postoperative recovery and increases the length of hospital stay, and also reduces the postoperative satisfaction of patients (10). Hence, the control of PONV is critical for the out-of-bed activity and functional rehabilitation of elderly patients.

Glucocorticoids exert anti-inflammatory effects by inhibiting the cyclooxygenase isoform 2 signaling pathway in the peripheral tissues and central nervous system, thereby reducing local and systemic inflammatory responses to control pain and nausea (11). Due to the potent antiemetic and anti-inflammatory properties, glucocorticoids are widely used in the perioperative management of gynecological surgery (12) and orthopedic surgery, including TKA (13–15), to reduce postoperative inflammatory responses, relieve postoperative pain, and prevent PONV.

Glucocorticoids are commonly used as prophylactic medications in the perioperative period of TKA to improve PONV (6, 16). In recent years, accumulating studies have shown that dexamethasone can alleviate PONV (17, 18). However, perioperative use of dexamethasone eventuates potential side effects, such as impeding wound healing and elevating blood glucose. Jiang et al. (19) reported that patients in the dexamethasone group had higher blood glucose levels in relative to the control group. As a long-acting glucocorticoid, betamethasone has smaller sodium and water retention effects and longer half-life than dexamethasone (20). Betamethasone has been applied in the perioperative period of breast surgery and cardiac surgery, and has achieved favorable results in controlling PONV (21–24). In orthopedic surgery, especially for patients undergoing primary TKA, the efficacy and safety of betamethasone in relieving PONV are still unknown. Therefore, this study aims to evaluate the efficacy and safety of preoperative prophylactic application of betamethasone for PONV in patients undergoing TKA through a prospective randomized controlled trial (RCT).

## 2 Materials and methods

### 2.1 Study design

This study was designed as a prospective RCT, which was reviewed and approved by the ethics committee of the Honghui Hospital Affiliated to Xi’an Jiaotong University (No.: 202305006) and registered in the Chinese Clinical Trial Registry (ChiCTR2300072532). All experimental procedures were designed and implemented according to the rules of the Declaration of Helsinki. All participating patients or their legal guardians signed written informed consent before surgery.

### 2.2 Inclusion and exclusion criteria of samples

Diagnostic criteria: According to the diagnostic criteria for knee osteoarthritis established by the American College of Rheumatology (ACR), which includes recurrent knee pain in the past month, the knee X-ray (in the standing or weight-bearing position) shows narrowing of the joint space, subchondral bone sclerosis and (or) cyst formation, and osteophyte formation at the edge of the joint; accompanied by any of the following (1) age ≥ 50 years; (2) stiffness of the affected knee ≤ 30 min; (3) crepitus (feeling) during activity. The severity of the disease is assessed using the Kellgren-Lawrence grading system for knee osteoarthritis, which is classified as Grade III/IV.

Inclusion criteria: Adult patients with knee osteoarthritis who met the diagnostic criteria of knee osteoarthritis and underwent primary unilateral TKA in Xi’an Honghui Hospital; adult patients with the American Society of Anesthesiologists physical status (ASA-PS) classification I-III; aged 50–80 years old; informed consent to participate in this study.

Exclusion criteria: Cardiovascular functional impairment (New York Heart Association classification > 2); renal dysfunction (glomerular filtration rate < 60 mL/min/1.73 m^2^); liver dysfunction (Child grade > B); poorly controlled diabetes mellitus (DM; HbA1C > 7.5%); allergic to any study drugs; taking any strong opioids in the past week and receiving systemic glucocorticoid treatment within 3 months before operation; alcohol dependence or drug abuse, such as opioid dependence (morphine, fentanyl, or oxycodone); cognitive impairment; the effect of psychological and neurological impairment on pain sensation; stopping intravenous (IV) patient-controlled analgesia; Body mass index (BMI) > 35 kg/m^2^; previous history of PONV/motion sickness; language barriers; unable or unwilling to provide informed consent or cannot comply with the trial requirements; all subjects considered by the investigator as unsuitable for participation in this study. The exclusion of patients will be implemented in cases where data loss or dropout occurs.

Termination criteria: Serious adverse reactions, as judged by the doctor, who should stop the clinical study of the case, i.e., terminate the clinical study of the case; Deterioration of the condition after treatment, as judged by the doctor, who should stop the clinical study of the case, i.e., terminate the clinical study of the case; Patients who do not want to continue the clinical study during the clinical trial process and request the doctor to terminate the clinical study of the case can terminate the clinical study of the case.

### 2.3 Experimental intervention

**FIGURE 1:**
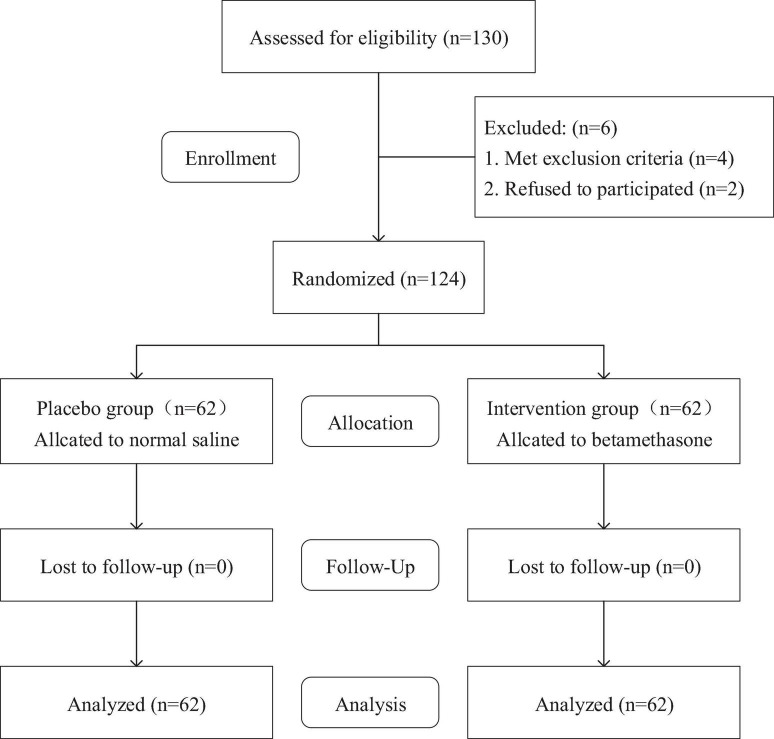
Flow chart of experimental design.

A total of 124 study patients were included according to the inclusion and exclusion criteria. The nurse used a computer-generated random number table to randomly assign the patients based on their admission order, with 62 patients in each group. The nurse placed the group assignment information of each patient in an opaque sealed envelope and brought it to the operating room on the morning of the surgery, and the nurse was not involved in this trial. After the patients signed the informed consent form, they were randomly assigned, and their group assignment was not disclosed to them until the results of the data analysis were obtained. Before surgery, a research assistant who was not involved in data collection prepared the drugs based on the patients’ sealed envelopes. The anesthesiologist injected the drugs before the induction of anesthesia. The control group received 2 mL normal saline 10 min before anesthesia induction; The experimental group received 2 mL (total dose: 10:52 mg) betamethasone sodium phosphate injection (Shandong Shenglu Pharmaceutical Co., Ltd; Shandong, China; 1 mL:5.26 mg) intravenously 10 min before anesthesia induction. All patients, surgeons, anesthesiologists, outcome assessors, data collectors, and statistical analysts were blind to the intervention (Figure 1).

### 2.4 Surgical methods

All patients in the two groups received general anesthesia, femoral nerve block, and pneumatic tourniquet on the affected limb. The conventional medial parapatellar approach was used to avoid the quadriceps tendon and expose the tibia and femur. Standard osteotomy was performed after clearing the proliferating synovial tissues and osteophytes in the joint cavity and releasing the ligaments. After the trial model prosthesis was installed, the balance of knee space in flexion and extension was checked and the patella was trimmed. Then, 20 ml of 0.2% ropivacaine was injected around the joint (Ruiyang Pharmaceutical Co., Ltd; Shandong, China; 10 mL: 100 mg). The whole joint cavity was immersed in type III amiloride and then rinsed with sterile normal saline pulse. After bone cement adhesion, the prosthesis was implanted and the tibial spacer was installed. After the bone cement was set, the joint cavity was rinsed with type III amiloride and sterile normal saline pulse again. Finally, the tourniquet was loosened and tranexamic acid was used for intraoperative hemostasis, followed by layer suture. No drainage tube was used in all included cases.

### 2.5 Postoperative nursing and rehabilitation schemes

The patients were sent to the ward after recovering from anesthesia and returned to a normal diet 6 h after being transferred to the ward. Ice compress was applied to the wound within 24 h after surgery, once every 3 h, 30 min each time. After 24 h of surgery, the patients were guided to perform knee joint range of motion exercise, quadriceps strength training, and machine passive assisted exercise, and received air pressure wave treatment to prevent thrombosis. Also, the patients were instructed to get out of bed and walk, and informed the personal precautions after surgery. All the training was carried out under the supervision and assistance of the physiotherapist. Patient-controlled analgesia (Dezocine, 0.5 mg/mL, 100 mL) (Dezocine Injection; Yangzi River Pharmaceutical Group Co., Ltd; Jiangsu, China; 1 mL:5 mg) with analgesic pump was applied postoperatively. Moreover, the patients was subcutaneously injected with low molecular weight heparin calcium injection 0.4 mL/d (Hebei Changshan Biochemical Pharmaceutical Co., Ltd; Hebei, China; 0.4 ml:4100axaiu) to prevent embolism postoperatively, which was maintained until discharge 3 days after surgery. After discharge, rivaroxaban tablets were orally administered at 2.5 mg/d (Qilu Pharmaceutical Co., Ltd; Shandong, China; 10 mg/tablet) for 10–14 days. The patients were instructed to continue to perform quadriceps strength exercise and knee range of motion exercise after discharge, and to accept review regularly until the knee function returned to normal. All patients had the same schemes to relieve postoperative pain and reduce PONV, and did not take other analgesic or antiemetic drugs during the trial.

### 2.6 Collection of experimental data

The baseline data of patients were collected after admission, including name, sex, age, height, weight, BMI, and smoking. The joint range of motion, fasting blood glucose, C-reactive protein (CRP), interleukin-6 (IL-6), and erythrocyte sedimentation rate (ESR) were recorded before surgery.

The primary indicators included the nausea severity and emesis episodes at 0, 2, 4, 6, 12, 24, and 48 h after surgery, and the antiemetic drugs required by patients within 48 h were counted. The severity of nausea was assessed by visual analogue scale (VAS), graded as no, mild, moderate, and severe nausea (VAS score 0 = no, 10 = the most severe nausea imaginable). In PONV treatment, when the patient had two or more PONV or severe nausea, 10 mg of metoclopramide hydrochloride injection (Shanghai Hefeng Pharmaceutical Co., Ltd; Shanghai, China; 1 mL:10 mg) was administered intramuscularly as the first-line antiemetic treatment. If nausea or emesis still existed after metoclopramide hydrochloride injection was administered twice within 30 min, 8 mg of ondansetron hydrochloride injection (Harbin Sanlian Pharmaceutical Co., Ltd; Heilongjiang, China; 4 ml:8 mg) was administered intramuscularly as the remedial treatment.

The secondary indicators were the pain scores in resting and active states at 6, 12, 24, and 48 h after surgery. VAS was used to assess the degree of pain (VAS score 0 = no pain, 10 = the most severe pain). When the patient reported severe pain (VAS > 6), oral administration of irecoxib tablets (Chengdu Shengdi Pharmaceutical Co., Ltd; Sichuan, China; 0.1 g/tablet) was given (0.1 g/time). Goniometer was used to measure the range of motion of knee flexion and extension 24 h after surgery. The laboratory test obtained the fasting blood glucose, CRP, IL-6, and ESR on the first and second postoperative days. In addition, the occurrence of postoperative adverse reactions (gastrointestinal hemorrhage and surgical site infection) was also recorded, as well as with the time required for the surgery.

### 2.7 Statistical analysis

In this study, the sample size was estimated through the data of 20 patients. For the 1:1 parallel control trial, under the assumption of the type I error rate = 5%, the sample size of 51 patients in each group was required to achieve 90% confidence. The estimated attrition rate was 20%, so we planned to recruit 65 patients in each group, with a total of 130 patients.

The data were input and processed using SPSS29.0 statistical software. The demographic data of two groups, including age, weight, height, and surgery time, were analyzed by the Mann-Whitney U test; the BMI was analyzed by the independent sample T test; gender and smoking status were analyzed by the chi-square test. The continuous data in clinical results, including emesis episodes, average nausea severity, consumption of antiemetics, pain scores, knee flexion and extension, and blood-related metrics were analyzed by the Mann-Whitney U test; the number of cases with severe nausea was statistically analyzed by rank sum test. A value of *P* < 0.05 indicated a significant difference.

## 3 Results

From June 2023 to March 2024, a total of 130 patients were recruited and planned to undergo initial unilateral TKA in our hospital. Among them, 4 patients were unqualified for this trial according to the exclusion criteria and 2 patients refused to participate in the trial. Finally, 124 patients were included in this trial. There were no statistically significant differences in baseline characteristics, smoking, and duration of surgery between the two groups (Table 1).

**TABLE 1:** Demographic data.

Variables	Placebo group (*n* = 62)	Intervention group (*n* = 62)	*P*-value
Age (year)	66.69 ± 5.816	68.74 ± 13.596	0.244
Sex (*n*, male/female)	22/40	21/41	0.850
Height (cm)	162.520 ± 7.602	161.400 ± 7.755	0.265
Weight (kg)	66.177 ± 9.983	67.030 ± 8.617	0.705
BMI (kg/m^2^)	25.049 ± 3.401	25.728 ± 2.789	0.226
Duration of surgery (min)	89.42 ± 13.725	87.71 ± 16.053	0.280
Smoking (*n*, yes/no)	10/52	8/54	0.610

BMI, body mass index; *P*-value indicates a significant difference among the groups.

The average number of emesis episodes was lower in the experimental group (0.060 ± 0.307) than that in the control group (0.390 ± 0.662) at 2 h after surgery, with a statistical difference (*P* < 0.001). No emesis occurred in both groups 24 h after surgery. There was no statistical difference in the total number of emesis episodes within 48 h after surgery between the two groups (*P* = 0.991). The postoperative use of metoclopramide in the experimental group (0.480 ± 2.163) was lower than that in the control group (4.520 ± 6.447), with a statistical difference between the two groups (*P* < 0.001) (Table 2).

**TABLE 2:** Mean number of emesis episodes of the two groups.

Parameters	Placebo group (*n* = 62)	Intervention group (*n* = 62)	*P*-value
**Number of emesis episodes**			
Postop 0 h	0.560 ± 1.223	0.180 ± 0.385	0.308
Postop 2 h	0.390 ± 0.662	0.060 ± 0.307	<0.001*
Postop 4 h	0.130 ± 0.424	0.050 ± 0.216	0.286
Postop 6 h	0.060 ± 0.307	0.000 ± 0.000	0.081
Postop 12 h	0.060 ± 0.307	0.000 ± 0.000	0.081
Postop 24 h	0.000 ± 0.000	0.000 ± 0.000	1.000
Postop 48 h	0.000 ± 0.000	0.000 ± 0.000	1.000
Total numbers	1.210 ± 9.525	0.290 ± 2.286	0.991
Metoclopramide (mg)	4.520 ± 6.447	0.480 ± 2.163	<0.001*

Postop, postoperative; Postop 0 h, time in the post-anesthesia care unit (PACU); total numbers, total numbers of emesis episodes during 48 h after surgery;

*statistical differences.

The average degree of nausea in the experimental group at 2, 4, 6, 12, and 24 h after surgery (0.630 ± 1.571, 0.260 ± 0.940, 0.030 ± 0.254, 0.000 ± 0.000, and 0.000 ± 0.000) was lower than that in the control group (2.290 ± 2.682, 1.440 ± 1.947, 0.840 ± 1.405, 0.470 ± 1.211, and 0.180 ± 0.713), with statistical differences (*P* < 0.001, *P* < 0.001, *P* < 0.001, *P* = 0.001, and *P* = 0.043). There was no statistical difference in the degree of nausea between the two groups at 0 h and 48 h after surgery (*P* = 0.060, *P* = 1.000). Neither the control group nor the experimental group developed nausea at 48 h after surgery (Table 3).

**TABLE 3:** Mean nausea severity in two groups.

Parameters	Placebo group (*n* = 62)	Intervention group (*n* = 62)	*P*-value
**Nausea severity**			
Postop 0 h	2.580 ± 3.139	1.600 ± 2.621	0.060
Postop 2 h	2.290 ± 2.682	0.630 ± 1.571	<0.001*
Postop 4 h	1.440 ± 1.947	0.260 ± 0.940	<0.001*
Postop 6 h	0.840 ± 1.405	0.030 ± 0.254	<0.001*
Postop 12 h	0.470 ± 1.211	0.000 ± 0.000	0.001*
Postop 24 h	0.180 ± 0.713	0.000 ± 0.000	0.043*
Postop 48 h	0.000 ± 0.000	0.000 ± 0.000	1.000

*Statistical differences.

In terms of the number of postoperative nausea severity, the average nausea degree of the experimental group at 2, 4, 6, 12, and 24 h after surgery was lower than that of the control group, with statistical differences (*P* < 0.001, *P* < 0.001, *P* < 0.001, *P* = 0.002, and *P* = 0.043) (Table 4).

**TABLE 4:** Number of nausea severity in two groups.

Parameters	Placebo group (*n* = 62)	Intervention group (*n* = 62)	*P*-value
Postop 0 h *n* (%)			0.068
No	35 (56.5%)	44 (71.0%)	
Mild	7 (11.3%)	7 (11.3%)	
Moderate	20 (32.3)	11 (17.7%)	
Severe	0 (0%)	0 (0%)	
Postop 2 h *n* (%)			<0.001*
No	34 (54.8%)	51 (82.3%)	
Mild	7 (11.3%)	8 (12.9)	
Moderate	21 (33.9)	3 (4.8%)	
Severe	0 (0%)	0 (0%)	
Postop 4 h *n* (%)			<0.001*
No	38 (61.3%)	57 (91.9%)	
Mild	17 (27.4%)	4 (6.5%)	
Moderate	7 (11.3%)	1 (1.6%)	
Severe	0 (0%)	0 (0%)	
Postop 6 h *n* (%)			<0.001*
No	46 (74.2%)	61 (98.4%)	
Mild	13 (21.0%)	1 (1.6%)	
Moderate	3 (3%)	0 (0%)	
Severe	0 (0%)	0 (0%)	
Postop 12 h *n* (%)			0.002*
No	53 (85.5%)	62 (100%)	
Mild	7 (11.3%)	0 (0%)	
Moderate	2 (3.2%)	0 (0%)	
Severe	0 (0%)	0 (0%)	
Postop 24 h *n* (%)			0.043*
No	58 (93.5%)	62 (100%)	
Mild	4 (6.5%)	0 (0%)	
Moderate + severe	0 (0%)	0 (0%)	
Postop 48 h *n* (%)			1.000
No	62 (100%)	62 (100%)	

*Statistical differences.

There was no statistical difference in pain scores between the two groups in the resting and active states (Table 5). There was no statistical difference between the experimental group and the control group in the knee range of motion before and after surgery (Table 6). There were no significant differences in blood glucose, CRP, IL-6, and ESR before surgery. In terms of blood glucose values, the experimental group and the control group did not show statistical differences after surgery (Table 7).

**TABLE 5:** Pain evaluation in two groups.

Parameters	Placebo group (*n* = 62)	Intervention group (*n* = 62)	*P*-value
**VAS at rest**			
Postop 6 h	1.660 ± 0.542	1.630 ± 0.520	0.773
Postop 12 h	1.690 ± 0.616	1.610 ± 0.491	0.585
Postop 24 h	1.820 ± 0.641	1.770 ± 0.612	0.638
Postop 48 h	1.900 ± 0.620	1.790 ± 0.681	0.254
**VAS with activity**			
Postop 6 h	4.790 ± 1.026	4.710 ± 0.733	0.597
Postop 12 h	4.900 ± 0.804	4.730 ± 0.705	0.218
Postop 24 h	5.000 ± 1.008	4.920 ± 0.893	0.328
Postop 48 h	4.850 ± 1.022	4.710 ± 0.837	0.154

VAS, visual analog score.

**TABLE 6:** Knee range of motion in two groups.

Parameters	Placebo group (*n* = 62)	Intervention group (*n* = 62)	*P*-value
**The difference between knee flexion angle and extension angle (°)**			
Preop	95.514 ± 13.071	94.840 ± 12.013	0.772
Postop 24 h	83.700 ± 8.217	86.400 ± 9.460	0.082

Preop, preoperative.

**TABLE 7:** Blood-related metrics in two groups.

Parameters	Placebo group (*n* = 62)	Intervention group (*n* = 62)	*P*-value
**Glucose (mmol/L)**			
Preop	5.607 ± 0.548	5.809 ± 0.728	0.206
Postop 1 d	7.465 ± 1.872	7.726 ± 1.646	0.132
Postop 2 d	6.433 ± 1.496	6.650 ± 1.656	0.669
**CRP level (mg/L)**			
Preop	2.310 ± 2.645	2.127 ± 2.107	0.531
Postop 1 d	20.720 ± 18.723	18.406 ± 19.637	0.364
Postop 2 d	65.235 ± 50.970	45.741 ± 47.044	0.014*
**IL-6 level (pg/mL)**			
Preop	1.582 ± 0.325	1.535 ± 0.381	0.097
Postop 1 d	79.477 ± 97.441	51.853 ± 67.202	0.039*
Postop 2 d	38.618 ± 36.282	25.143 ± 31.912	0.006*
**ESR (mm/h)**			
Preop	10.967 ± 9.175	13.419 ± 12.377	0.402
Postop 1 d	17.903 ± 13.667	20.016 ± 12.506	0.129
Postop 2 d	27.403 ± 14.260	28.483 ± 15.468	0.638

CRP, C-reactive protein; IL-6 level, interleukin-6; ESR, erythrocyte sedimentation rate.

*Statistical differences.

CRP in the experimental group on the second day after surgery (45.741 ± 47.044) was lower than that in the control group (65.235 ± 50.970), with a statistical difference (*P* = 0.014). IL-6 was lower in the experimental group on the first day after surgery (51.853 ± 67.202) and the second day after surgery (25.143 ± 31.912) than that in the control group on the first day after surgery (79.477 ± 97.441) and the second day after surgery (38.618 ± 36.282), with statistical differences (*P* = 0.039, *P* = 0.006) (Table 7).

No adverse reactions of surgical site infection and gastrointestinal hemorrhage occurred in the two groups of patients during clinical observation (Table 8).

**TABLE 8:** Postoperative adverse reactions occurred in both groups.

Parameters	Placebo group (*n* = 62)	Intervention group (*n* = 62)	*P*-value
Surgical site infection (*n*)	0 (0%)	0 (0%)	1.000
Gastrointestinal hemorrhage (*n*)	0 (0%)	0 (0%)	1.000

## 4 Discussion

PONV refers to nausea, vomiting, or retching occurring within 24 h after surgery. The occurrence of PONV is closely related to the vomiting center and affected by the patient’s own situation, the use of drugs, and anesthesia surgery. Statistics estimate that the incidence of PONV is 25–30%, and particularly major surgery and high-risk patients are prone to PONV (25). The development of enhanced recovery after surgery (ERAS) aims to reduce the incidence of stress reactions and complications in perioperative patients, shorten the length of hospital stay, and accelerate the rehabilitation of patients (26). PONV is a common complication post TKA. Despite many antiemetic methods, PONV is closely related to postoperative dissatisfaction of patients receiving TKA (27–29).

The mechanism of PONV is complex, involving central and peripheral receptors and multiple nerve conduction pathways (25). After the receptors of the digestive system, cerebral cortex, or vestibular organs are stimulated, the afferent signals are transmitted to the vomiting center through the vagus nerve, sympathetic nerve, glossopharyngeal nerve, etc. In addition, various toxins, metabolites, or drugs in human blood and cerebrospinal fluid can directly stimulate dopamine receptors, 5-HT3 receptors, histamine receptors, cholinergic receptors, etc., resulting in excitatory signals projecting to the emetic chemosensory central area on the ventral side of the fourth ventricle of the medulla oblongata. After the integration of the nerve center, stimuli are sent to the effectors via the vagus nerve, sympathetic nerve, trigeminal nerve, glossopharyngeal nerve, hypoglossal nerve, spinal nerve, etc., resulting in relaxation of the sphincter in the upper esophagus and contraction of the diaphragm and abdominal muscles, which compresses the stomach to increase its pressure. Thus, the contents of the stomach are expelled from the body through the esophagus and oral cavity, that is, vomiting occurs (30).

Due to the complex mechanism of PONV, there are multiple drugs for the prevention and treatment of PONV. Glucocorticoid is one of the regimens to treat PONV. Glucocorticoids can reduce prostaglandin synthesis and endogenous opioid release, thereby relieving PONV through its central antiemetic action (31, 32). Betamethasone is a potent and long-acting glucocorticoid with anti-inflammatory and immunosuppressive properties. As a isomer of dexamethasone, the effect of betamethasone is equivalent to that of dexamethasone, but its sodium and water retention effects are smaller than dexamethasone, and its efficacy is also slightly longer. Therefore, betamethasone is usually combined with other drugs to make mixed preparations (20, 33).

In patients undergoing TKA, PONV occurs due to anesthetics, surgical trauma, and analgesic drugs, all of which can lead to postoperative gastrointestinal reactions (34). Meanwhile, surgical trauma leads to immune cell aggregation, which can release different systemic inflammatory biomarkers and increase the occurrence of PONV (8, 9, 35). In this study, there were significant differences in PONV between the two groups. The nausea severity of the betamethasone group at 2, 4, 6, 12, and 24 h after surgery was significantly lower than that of the control group, and the consumption of metoclopramide was also less than that of the control group (*P* < 0.001). In addition, the average number of emesis episodes in the betamethasone group was also lower than that in the control group 2 h after surgery. Our results are consistent with the findings of Champion et al. (21) in patients undergoing cardiac surgery, which proves that betamethasone can effectively reduce the occurrence of PONV. Notably, we found that there was no statistical difference in the incidence of PONV between the two groups during the recovery process in the postanesthesia care unit (PACU), which may be related to the slow antiemetic effect of betamethasone. This result is consistent with the conclusion of Olanders et al. (23) that betamethasone has an effect on nausea and vomiting within 4–12 h after surgery. Four patients (6.5%) in the blank group still had mild nausea at 24 h after surgery, but not in the experimental group, indicating that the long-term mechanism of betamethasone can reach 24 h after surgery, which was also similar to the findings of Olanders et al. (23). However, the incidence of PONV at 48 h after surgery was similar in the two groups, which may be related to the systemic absorption of betamethasone and the metabolism of narcotic drugs (36).

Glucocorticoids exert potent anti-inflammatory effects by inhibiting phospholipase A2 to reduce the production of cyclooxygenase and lipoxygenase pathway products, thereby repressing systemic inflammation, pain, and acute stress response (31). In our study, although the betamethasone group had lower pain scores than the control group at 48 h after surgery, the difference did not reach statistical significance. This finding conflicts somewhat with other studies of glucocorticoids, in which more convincing antiemetic and analgesic effects have been observed. The lack of statistical difference in pain scores between the two groups may be the result of multiple effects of drug injection route, small drug dose (37), small sample size and perioperative multimodal analgesia, which are similar to reports in other surgeries (3, 23). In the study of Li (35), the results showed that intravenous glucocorticoids were more effective in reducing blood inflammatory biomarkers, while local glucocorticoids had better clinical outcomes in postoperative pain management. In this trial, pain was a secondary outcome measure, and multimodal pain control was specified to protect patients from severe pain. In perioperative pain studies, minimal clinically important differences (MCID) (38) may be used to measure outcomes after the use of an analgesic intervention. The clinical significance of improvement, characterized by pain relief or attainment of patient satisfaction, outweighs the mere statistical significance. CRP and IL-6, as markers of acute inflammation, were elevated after surgery. However, the levels of IL-6 and CRP on the second day after surgery in the betamethasone group were lower than those in the control group, indicating the effectiveness of glucocorticoids for systemic inflammation, which is consistent with the clinical observation of glucocorticoids reported in other studies (9, 35, 36). In the study of Xu et al. (9), two intravenous injections of 10 mg dexamethasone had analgesic effect and reduced the incidence of PONV, and the levels of CRP and IL-6 at 24, 48, and 72 h after surgery were significantly reduced.

In this study, the blood glucose levels between the two groups did not show statistical differences, suggesting that the use of betamethasone did not lead to dramatic changes in blood glucose levels after surgery. Arumugam et al. (39) demonstrated that the blood glucose of patients did not increase significantly after the use of 4 and 8 mg dexamethasone. In the study of Lei et al. (37), there was no difference in blood glucose levels between groups of patients with intravenous dexamethasone in TKA. Chen et al. (15) found that there was no statistically significant difference in blood glucose levels between the groups on the 1st, 2nd, and 3rd postoperative days after the perioperative use of dexamethasone. This study excluded DM patients with poor glycemic control (HbA1C > 7.5%), and the results also proved that preoperative prophylactic medication of betamethasone did not affect the blood glucose levels of patients.

No glucocorticoid-related complications such as wound infection and gastrointestinal hemorrhage occurred in the two groups of patients in this study. Segelman et al. (24) applied a single dose of betamethasone to the patients receiving knee arthroscopy in the outpatient department, and followed up the patients for 3 months. It was found that the patients who received betamethasone did not develop adverse events, while 6 patients who received placebo had adverse events. In the study of Wu et al. (16), dexamethasone was used at low doses for many times during the perioperative period, and no patient had obvious adverse reactions and/or complications during the postoperative follow-up. The clinical trial of Xu et al. (9) showed that perioperative administration of double low-dose dexamethasone to patients receiving TKA did not increase the risk of early surgical wound infection and gastrointestinal hemorrhage. The results of Chen et al. (15) showed that the use of dexamethasone in the perioperative period of TKA did not cause surgical site infection and gastrointestinal hemorrhage in patients during hospitalization and 6-week follow-up. Similar to dexamethasone, our results also illustrate the safety of betamethasone in the perioperative period of TKA.

There are some limitations in this study. First, the analgesic pump containing opioids is a common cause of nausea and vomiting. There are individual differences in the nausea response to the analgesic pump, which may affect the experimental results. Second, there is a lack of long-term follow-up. Hence, the long-term safety of betamethasone is still unknown. Finally, all participants in this study are recruited from the same medical center, which is an orthopedic specialist hospital. Therefore, the results may not be applicable to different type of medical institutions worldwide, multi-center research is needed to further enhance the reliability of the results.

## 5 Conclusion

In conclusion, our prospective RCT proves that perioperative prophylactic application of betamethasone has favorable efficacy and safety for the management of PONV in patients receiving TKA, providing a novel scheme for the prophylactic treatment of nausea and vomiting after TKA in the future.

## Data Availability

The original contributions presented in this study are included in this article/supplementary material, further inquiries can be directed to the corresponding authors.
